# Characterization of Yeast Extracellular Vesicles: Evidence for the Participation of Different Pathways of Cellular Traffic in Vesicle Biogenesis

**DOI:** 10.1371/journal.pone.0011113

**Published:** 2010-06-14

**Authors:** Débora L. Oliveira, Ernesto S. Nakayasu, Luna S. Joffe, Allan J. Guimarães, Tiago J. P. Sobreira, Joshua D. Nosanchuk, Radames J. B. Cordero, Susana Frases, Arturo Casadevall, Igor C. Almeida, Leonardo Nimrichter, Marcio L. Rodrigues

**Affiliations:** 1 Laboratório de Estudos Integrados em Bioquímica Microbiana, Instituto de Microbiologia Professor Paulo de Góes, Rio de Janeiro, Rio de Janeiro, Brazil; 2 Department of Biological Sciences, The Border Biomedical Research Center, University of Texas at El Paso, El Paso, Texas, United States of America; 3 Department of Medicine, Albert Einstein College of Medicine, Bronx, New York, United States of America; 4 Laboratory of Genetics and Molecular Cardiology, Heart Institute (InCor), University of São Paulo, São Paulo, São Paulo, Brazil; 5 Department of Microbiology and Immunology, Albert Einstein College of Medicine, Bronx, New York, United States of America; 6 Laboratório de Biotecnologia, Instituto Nacional de Metrologia, Normalização e Qualidade Industrial, Rio de Janeiro, Rio de Janeiro, Brazil; National Institutes of Health, United States of America

## Abstract

**Background:**

Extracellular vesicles in yeast cells are involved in the molecular traffic across the cell wall. In yeast pathogens, these vesicles have been implicated in the transport of proteins, lipids, polysaccharide and pigments to the extracellular space. Cellular pathways required for the biogenesis of yeast extracellular vesicles are largely unknown.

**Methodology/Principal Findings:**

We characterized extracellular vesicle production in wild type (WT) and mutant strains of the model yeast *Saccharomyces cerevisiae* using transmission electron microscopy in combination with light scattering analysis, lipid extraction and proteomics. WT cells and mutants with defective expression of Sec4p, a secretory vesicle-associated Rab GTPase essential for Golgi-derived exocytosis, or Snf7p, which is involved in multivesicular body (MVB) formation, were analyzed in parallel. Bilayered vesicles with diameters at the 100–300 nm range were found in extracellular fractions from yeast cultures. Proteomic analysis of vesicular fractions from the cells aforementioned and additional mutants with defects in conventional secretion pathways (*sec1-1*, fusion of Golgi-derived exocytic vesicles with the plasma membrane; *bos1-1*, vesicle targeting to the Golgi complex) or MVB functionality (*vps23*, late endosomal trafficking) revealed a complex and interrelated protein collection. Semi-quantitative analysis of protein abundance revealed that mutations in both MVB- and Golgi-derived pathways affected the composition of yeast extracellular vesicles, but none abrogated vesicle production. Lipid analysis revealed that mutants with defects in Golgi-related components of the secretory pathway had slower vesicle release kinetics, as inferred from intracellular accumulation of sterols and reduced detection of these lipids in vesicle fractions in comparison with WT cells.

**Conclusions/Significance:**

Our results suggest that both conventional and unconventional pathways of secretion are required for biogenesis of extracellular vesicles, which demonstrate the complexity of this process in the biology of yeast cells.

## Introduction

Protein secretion is a complex process that involves many organelles and accessory molecules. In eukaryotic cells, the most well-studied pathway of protein secretion involves vesicular migration from the endoplasmic reticulum to the *trans* face of the Golgi and then loading into a complex network of vesicles, the *trans-*Golgi reticulum [1]. The vesicular post-Golgi network is the most prominent example of conventional mechanism of protein secretion. These proteins are sorted in the *trans-*Golgi network into transport vesicles that immediately move to and fuse with the plasma membrane, releasing their contents by exocytosis [1]. There are multiple unconventional mechanisms of protein secretion in eukaryotes [2]. One of these mechanisms requires the formation of the exosomes, which are vesicles derived from membrane invagination within endocytic compartments (endosomes). The formation of internal vesicles in the lumen of endosomes generates the multivesicular bodies (MVBs), which can fuse with the plasma membrane, resulting in the release of internal vesicles to the extracellular milieu as exosomes [3]. Extracellular vesicle formation could also require other cellular pathways, as suggested for *Dictyostelium discoideum*. In this organism, it has been hypothesized that the Golgi reassembly stacking protein (GRASP), which is attached peripherally to the cytoplasmic surface of Golgi membranes, is required for the vesicular release of acyl-CoA binding protein [4].

Fungal cells secrete molecules of different chemical natures and molecular masses. As typical eukaryotic organisms, fungal cells use conventional pathways of secretion involving post-Golgi vesicles that fuse with the plasma membrane to release their cargo [5], [6]. In fact, it is well known that yeast cells continuously secrete a number of enzymes that remain localized in the periplasm [7] but, until recently, *trans-*cell wall secretion in fungi is a relatively unknown cellular event, which consequently, had not been extensively studied. Over the past few years, the existence of *trans*-cell wall transport of intact vesicles has been reported and partially characterized in pathogenic and non-pathogenic species of fungi [8], [9], [10], [11], [12], [13], [14], [15]. These vesicles contain a number of proteins, lipids, polysaccharides and pigments of a wide molecular mass range [8], [11], [14], [15]. Therefore, we have proposed that extracellular vesicle secretion represents a eukaryotic solution to the problem of *trans-*cell wall transport, especially for large molecules [9], [12].

Although fungal extracellular vesicles have been generally termed ‘fungal exosomes’ [9], [14], [16], different studies suggest that their release to the extracellular space requires elements of the conventional post-Golgi secretory pathway [16], [17]. Yoneda and Doering demonstrated that a *Cryptococcus neoformans* strain defective in the production of Sav1p, a homolog of the *S. cerevisiae* small GTPase Sec4p, accumulates post-Golgi vesicles under restrictive conditions [17], a morphological feature that was initially described for *S. cerevisiae* [6], [18], [19]. More precisely, the *sav1* mutant of *C. neoformans* had defective protein secretion and accumulated exocytic vesicles at the septum and the bud during cell division. Remarkably, these vesicles also contained a polysaccharide destined to the extracellular space, suggesting that post-Golgi secretion is involved with the transfer of macromolecules through the cell wall. These findings were further supported by an independent study showing that exposure of yeast cells to brefeldin A, which interferes with the retrograde protein transport from the Golgi apparatus to the endoplasmic reticulum, results in the inhibition of polysaccharide assembly at the outer layer of the *C. neoformans* cell wall [20]. In agreement with these observations, a *C. neoformans* RNAi mutant strain lacking expression of Sec6p, an 88 kDa subunit of the exocytic complex that mediates polarized targeting of secretory vesicles to active sites of exocytosis, was unable to produce extracellular vesicles [16].

Although the studies mentioned above suggested the involvement of conventional secretory mechanisms in the vesicular export of polysaccharides in *C. neoformans*, it remained unclear whether extracellular polysaccharides were targeted to the cell surface exclusively in post-Golgi vesicles or via recycling endosomes [17]. Hence, the possibility that that the post-Golgi polysaccharide-containing vesicles are sorted to a compartment other than the plasma membrane, such as the late endosomes and the MVBs cannot be ruled out. In fact, endosomes and MVBs can be connected to the *trans-*Golgi secretory pathway [3], thus both pathways could be involved in vesicular polysaccharide export in fungi. The molecular machinery implied in MVB formation and sorting is widely known in *S. cerevisiae* [21], but studies have not evaluated extracellular vesicle transport and the connection to exosomes.

In this study, we characterized extracellular vesicles produced by the model yeast *S. cerevisiae*. Based on recent suggestions that fungal extracellular vesicles resemble exosomes but originate from Golgi-related pathways [9], [14], [16], we also characterized vesicle fractions from culture supernatants of mutants with defects in MVB formation and post-Golgi secretion. Our results indicate that mutations in both pathways affect vesicle composition. Nevertheless, yeast cells mutated in the *SEC4* gene, previously shown to accumulate cytoplasmic vesicles [19], were defective in the secretion of vesicles to the extracellular space, suggesting a key role for the Golgi-derived secretory pathway in the *trans-*cell wall traffic in yeast cells.

## Materials and Methods

### Strains

The *S. cerevisiae* strains used in this study included RSY255, RSY113, SEY6210 and BY4741 wild type (WT) cells and several yeast secretory mutants, as subsequently described in this section and summarized in Table 1. Strains RSY782, SF2642-1D, and RSY954 are respectively, temperature sensitive *sec1-1*, *sec4-2* and *bos1-1* (also known as *sec32-1*) mutants. Strains EEY6-2 and EEY9 correspond to mutant cells lacking expression of Vps23p and Snf7p (also known as Vps32p), respectively. Strain GRH1delta is defective in the expression of GRASP. WT and mutant cells were cultivated in Sabouraud dextrose broth for 24 h at 25°C with shaking. Based on recent literature, strains SF2642-1D (*sec4-2*) and EEY9 (*snf7*) were used as prototypes of yeast cells defective in post-Golgi or endosome-dependent vesicular secretion, respectively [17], [22]. Therefore, except for proteomic analyses where all strains listed above were used, experiments in this study were performed using *sec4* and *snf7* mutants and related WT strains.

**Table 1:** Yeast strains.

WT strain	Mutation (mutant strain)	Cellular event affected by mutation	Origin	Reference
RSY255	*sec1-1* (RSY782)	Membrane fusion*	Schekman laboratory	[63]
RSY113	*sec4-2* (SF2642-1D)	Vesicle targeting to the cell surface*	Schekman laboratory	[32]
RSY255	*bos1-1*, also known as *sec32.2* (RSY954)	Vesicle targeting to the Golgi complex*	Schekman laboratory	[47]
SEY6210	*snf7*, also known as *vps32* (EEY9)	Vesicle invagination within multivesicular bodies	Emr laboratory	[22], [31]
SEY6210	*vps23* (EEY6-2)	Late endosomal trafficking	Emr laboratory	[46]
BY4741	*grh1*, also known as *grasp* (GRH1delta)	Unconventional secretion of acyl coenzyme A–binding protein	EUROSCARF/Malhotra laboratory	[39]

(*) Phenotype observed under restrictive temperature.

### Vesicle isolation

Extracellular vesicles from strains SEY6210 and BY4741 and corresponding *vps* and *grasp* mutants were isolated from culture supernatants, using variations of methods previously described [8], [14], [15]. For vesicle isolation from cultures of strains RSY255 and RSY113 and the corresponding *sec* mutants, supernatants were removed from 24 h cultures, cells were washed three times, and fresh medium was added for further incubation at 37°C for 1 to 18 h at which time supernatants were removed and processed for vesicle isolation. Cell viability was similar in WT and mutant cultures, as determined by colony forming unit counting (data not shown). For all cultures, yeast cells and debris were removed by sequential centrifugation at 4,000 and 15,000 *g* (15 min, 4°C) [8], [14], [15]. Supernatants were collected and concentrated by approximately 20-fold using an Amicon ultrafiltration system (cutoff  = 100 kDa). The concentrate was again centrifuged at 4,000 and 15,000 *g* (15 min, 4°C) and passed through filtering membranes (0.8 µm pores). Filtered fractions were finally centrifuged at 100,000 *g* for 1 h at 4°C. Pellets were then washed by three sequential suspension and centrifugation steps, each consisting of 100,000 *g* for 1 h at 4°C with 0.1 M Tris buffered saline (TBS, pH 7.4). The resulting pellets were then suspended in fixative solution for electron microscopy analysis or prepared for lipid and protein determinations, as described below. To avoid the possibility of artifactual isolation of intracellular organelles from dead cells, similar protocols were used with suspensions of heat-killed cells instead of living yeast as described previously [15]. Vesicle-like structures were not observed under these conditions.

### Transmission electron microscopy (TEM)

Pellets obtained after centrifugation of cell-free supernatants at 100,000 *g* were fixed with 2% glutaraldehyde in 0.1 M cacodylate at room temperature for 2 h, and then incubated overnight in 4% formaldehyde, 1% glutaraldehyde, and 0.1% PBS. The samples were incubated for 90 min in 2% osmium, serially dehydrated in ethanol, and embedded in Spurrs epoxy resin. Thin sections were obtained on a Reichart Ultracut UCT and stained with 0.5% uranyl acetate and 0.5% lead citrate. Samples were observed in a JEOL 1200EX transmission electron microscope operating at 80 kV.

### Light scattering analysis of vesicles

Vesicles undergo Brownian motion that translates into light scattering fluctuations in a liquid phase. This property can be measured by dynamic light scattering (DLS) techniques providing information on the size and heterogeneity of the sample [11]. Measurement of vesicle size by DLS was performed in a 90Plus/BI-MAS Multi Angle Particle Sizing analyzer (Brookhaven Instruments) as described recently by Einsenman and colleagues [11].

### Sterol analysis

Sterols are structural components of fungal extracellular vesicles and markers of vesicle membranes [15]. For this reason, these molecules were used in our model as molecular markers of vesicular secretion in indirect quantification of vesicle fractions. Sterols were also analyzed in cellular fractions, for comparative analyses in the different strains used in this study. Intact cells were collected by centrifugation and washed with PBS. These pellets or vesicle fractions were first suspended in methanol and then two volumes of chloroform were added. The mixture was vigorously vortexed and centrifuged to discard precipitates, dried by vacuum centrifugation and partitioned according to [23]. The lower phase, containing neutral lipids, was recovered for analysis by high performance thin layer chromatography (HPTLC). The lipid extract was loaded into HPTLC silica plates (Si 60F254s, LiChrospher, Merck, Germany) and separated using a solvent system containing hexane:ether:acetic acid (80∶40∶2, v/v/v). The plate was sprayed with a solution of 50 mg ferric chloride (FeCl_3_) in a mixture of 90 ml water, 5 ml acetic acid and 5 ml sulfuric acid. After heating at 100°C for 3–5 min, the sterol spots were identified by the appearance of a red-violet color. Stained HPTLC plates were digitalized using Adobe Photoshop CS (version 8.0) and densitometrically analyzed with the Scion Image software (version Alpha 4.0.3.2). Sterol content was also evaluated using the quantitative fluorimetric kit “Amplex Red Sterol Assay Kit” (Molecular Probes). Sensitivity of sterol detection in this test is approximately 8 ng in intact membranes, with no requirement of lipid extraction. Vesicle pellets were suspended in 500 µl PBS and 10% of the sample was evaluated in this assay according to manufacturer's instructions. In all analyses, sterol content in each fraction was normalized to the number of viable cells in yeast cultures.

### Protein identification by liquid chromatography-tandem mass spectrometry (LC-MS/MS)

Vesicle proteomics followed the methodology recently established for the analysis of extracellular vesicle fractions from fungal cells [8], [14]. Briefly, purified vesicles were suspended in 40 µl 400 mM NH_4_HCO_3_, containing 8 M urea, and the disulfide bonds were reduced by the addition of 10 µl 50 mM dithiotreitol, followed by incubation for 15 min at 50°C. Cysteine residues were alkylated by the addition of 100 mM iodoacetamide (10 µl), followed by incubation for 15 min at room temperature under protection from the light. The final concentration of urea was then adjusted to 1 M and the mixture was supplemented with 4 µg sequencing-grade trypsin (Promega) for overnight digestion at 37°C. Resulting tryptic peptides were desalted in C18 reverse-phase in-house ziptip columns (POROS R2 50, Applied Biosystems), as described by Jurado et al. [24]. Samples were finally redissolved in 5% acetonitrile (ACN), containing 0.5% formic acid (FA), and loaded onto a C18-trap column. The separation was performed on a capillary reverse-phase column connected to a nanoHPLC system (nanoLC 1D Plus, Eksigent). Peptides were eluted in a linear gradient (5–40%) of solvent B (solvent A: 5% ACN/0.1% FA; solvent B: 80% ACN/0.1% FA) during 200 min and directly analyzed in an electrospray-linear ion trap-mass spectrometer (LTQ XL/ETD, Thermo Fisher) equipped at the front end with a Triversa NanoMate nanospray source (Advion). The nanospray was set at 1.35 kV and 0.2 psi N_2_ pressure using a chip A (Advion). MS spectra were collected in centroid mode at the 400–1700 *m/z* range and the ten most abundant ions were subjected twice to collision induced dissociation (CID) with 35% normalized collision energy, before being dynamically excluded for 60 sec.

All MS/MS spectra from peptides with 800–3500 Da, more than 10 counts, and at least 15 fragments were converted into DTA files using Bioworks v.3.3.1 (Thermo Fisher). DTA files were submitted to database search using TurboSequest [25] and the *S. cerevisiae* protein database (downloaded on June 8^th^, 2008 from www.yeastgenome.org) combined with human keratin and porcine trypsin sequences (downloaded on June 8^th^, 2008 from www.ncbi.nlm.nih.gov/protein). All the sequences were used in the correct and reverse orientations, forming a database with 13,760 entries. The database search parameters included: i) trypsin cleavage in both peptide termini with one missed cleavage site allowed; ii) carbamidomethylation of cysteine residues as a fixed modification; iii) oxidation of methionine residues as a variable modification; and iv) 2.0 Da and 1.0 Da for peptide and fragment mass tolerance, respectively. TurboSequest outputs were filtered with DCn ≥0.05, peptide probability ≤0.05, and Xcorr ≥1.5, 2.0, and 2.5 for singly-, doubly-, and triply-charged peptides, respectively. After filtering, the files were exported into XML formats and the peptides sequences were assembled into proteins using an in-house written script (Nakayasu, Sobreira, and Almeida, unpublished data). The protein hits were re-filtered with sum of peptide Xcorr ≥3.5. The false-discovery rate (FDR) was estimated as described previously [8], [14]. Proteins with shared peptides were assembled into groups to assess the redundancy issue. For semi-quantitative calculations, another in-house script was elaborated to combine respective peptides and spectral counts into their respective protein groups (Nakayasu, Sobreira, and Almeida, unpublished data). Semi-quantitative determinations of protein abundance in each sample were based on the calculation of the *e*xponentially *m*odified *p*rotein *a*bundance *i*ndex (emPAI), according to the methodology proposed by Ishihama et al. [26]. emPAI Data were further validated by the methodology described by Liu et al. [27], using the number of spectra acquired for each protein (spectral count). The relevant differences in protein abundance were selected when a two-fold incremental change variation (WT vs. mutant proteins) after spectral count and emPAI calculation was observed. A total of 400 proteins were identified; however, those (n = 273) with less than 10 spectra after MS/MS analysis of peptides were excluded from the spectral count analysis. Using this approach we could semi-quantify 127 proteins. This group of proteins was used in the analyses detailed in Tables S1 and S2 and related data. The whole set of results is available in Tables S3, S4 and S5.

### Bioinformatics

Venn diagrams were prepared using the Venny tool, available at http://bioinfogp.cnb.csic.es/tools/venny/index.html. Functional networks in vesicle proteins were prepared using the Osprey Network Visualization software (version 1.2.0), with the *S. cerevisiae* database (available from the software). Analysis of the putative presence of glycosylphosphatidylinositol (GPI)-anchored sequences in vesicle proteins was performed as described recently [28], using the GPI-anchored protein prediction program FragAnchor (http://navet.ics.hawaii.edu/~fraganchor/NNHMM/NNHMM.html) [29]. Correlation graphs and *R* ^2^ calculations were performed with GraphPad Prism version 5.00 for Windows, GraphPad Software, San Diego California USA, www.graphpad.com. The presence of signal peptide cleavage sites in amino acid sequences from vesicle proteins was predicted with the SignalP 3.0 Server (http://www.cbs.dtu.dk/services/SignalP/), as previously described [30].

## Results

### Morphological features and diameter distribution of *S. cerevisiae* vesicles

Vesicle morphology was analyzed in both WT and mutant *S. cerevisiae* cells. The selection of mutant strains for this analysis was based on the hypothesis that fungal extracellular vesicles derive from MVB-related pathways for exosome formation or from post-Golgi conventional secretion [9], [14], [16], [17]. In this context, our analyses included the *snf7* mutant, which shows defective MVB formation [31], and the *sec4-2* mutant, due to the implication of the *SEC4* gene in the secretion of Golgi-derived vesicles in yeast cells [17], [32].

**Figure 1:**
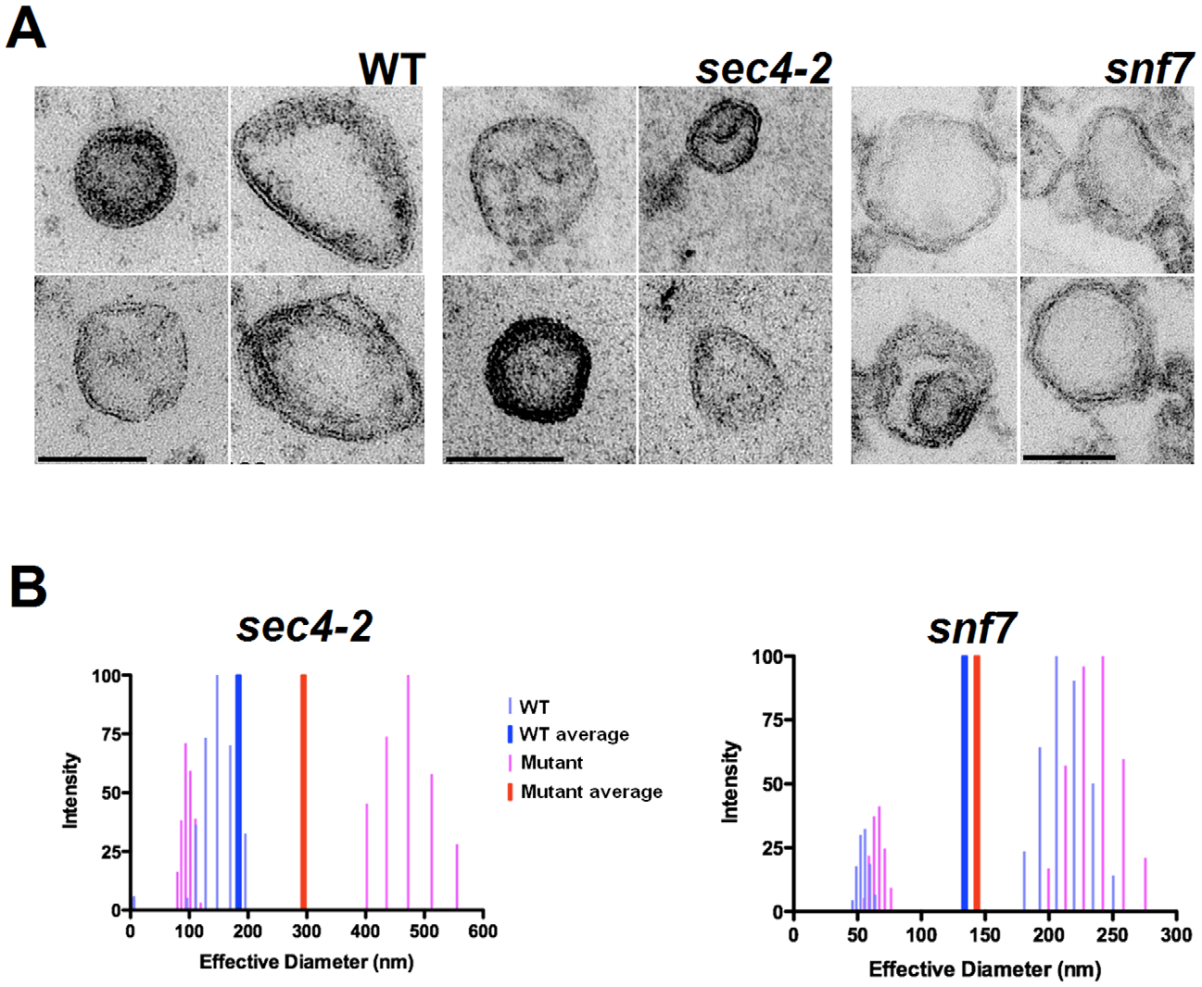
Morphology and diameter of *S. cerevisiae* extracellular vesicles. A. TEM of vesicles isolated from WT (WT) and mutant cells. Each individual panel exemplifies the typical vesicle morphology for the cell group specified in the top. WT fractions shown in left panels were obtained from strain RSY113, parental of the *sec4-2* mutant. Similar morphological features (not shown) were observed in vesicle fractions obtained from strain SEY6210 (parental of the *snf7* mutant). Scale bar, 100 nm. B. Light scattering analysis showing diameter distribution and average values of vesicles obtained from WT (WT) or mutant (*sec4-2* and *snf7*) cells.

Morphological analysis of yeast extracellular vesicles by TEM revealed bilayered structures with varying levels of electron density (Figure 1A). The vesicles were generally round or ovoid and sometimes resembled multivesicular structures. No morphologic alterations were apparent in vesicles obtained from *sec4-2* and *snf7* mutants. Analysis of the effective diameter of vesicle fractions obtained from WT cells revealed average values ranging from 133.9±4.2 (strain SEY6210, parental of *snf7*) to 183.9±24.0 (strain RSY113, parental of *sec4-2*) nm (Figure 1B). In interpreting these numbers, and the results below, it is noteworthy that dynamic light scattering tends to overestimate vesicle diameter relative to other techniques (for discussion on this effect see [11]). However, for the purposes of this study we have used dimensions obtained for dynamic light scattering for all comparisons thus controlling for any systematic trends in vesicle size inherent to the technique. Vesicles from strain SEY6210 were clearly distributed in two different populations, ranging from 50 to 75 and 180 to 250 nm in effective diameter. Mutation in the *SNF7* gene had little or no influence in vesicle diameter, which shifted from 133.9±4.2 nm in the vesicle fraction from WT cells to 143.5±2.1 nm for vesicles from the mutant cells. The two-population profile of distribution of vesicle diameter in fractions from the *snf7* mutant was very similar to that found in vesicles from the parental strain SEY6210. On the other hand, mutation in the *SEC4* gene significantly affected the diameter of extracellular vesicles. While most of the vesicles from WT cells (strain RSY113) were in the range of 100 to 200 nm, *sec4-2* vesicles were distributed in two populations. The mutant vesicles were distributed in either a population ranging from 80 to 120 nm or in a population with very high diameters (400–550 nm). Average values shifted from 183.9±24.0 nm (RSY113 strain) to 294.5±117.9 nm (*sec4-2* mutant). Hence, a *SEC4* mutation resulted in a qualitative difference in the vesicles produced by *S. cerevisiae*.

### Proteomic analysis reveals a complex composition in *S. cerevisiae* extracellular vesicles

**Figure 2:**
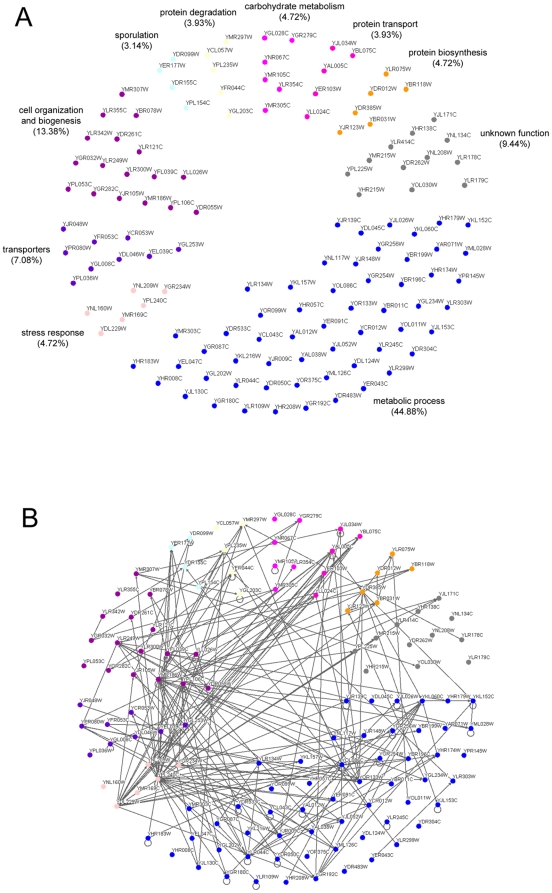
Functional distribution of proteins in extracellular vesicle fractions obtained from *S. cerevisiae* WT cells. A. Proteins were grouped by color as indicated according to their function in the cellular metabolism. B. Functional interrelationship in the collection of vesicular proteins. For protein identification according with each individual code, see Table S1.

**Figure 3:**
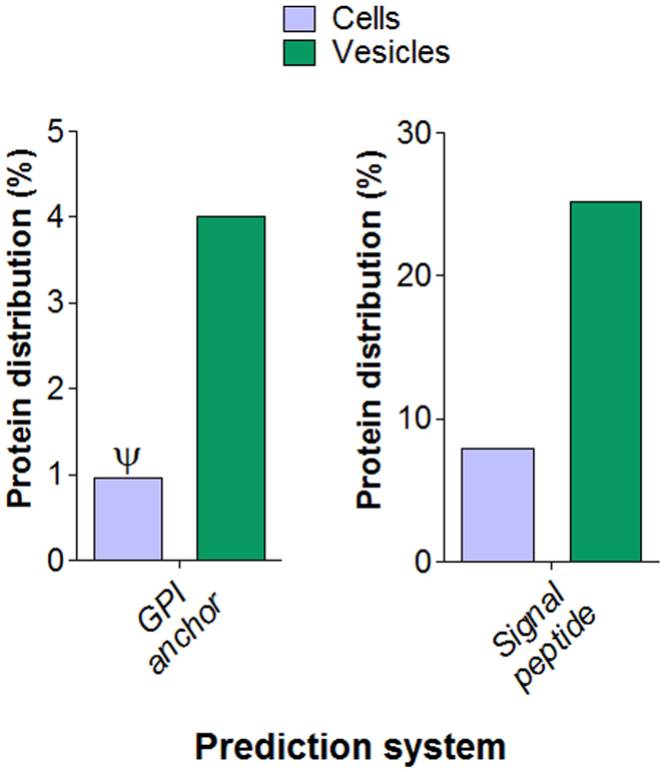
Prediction of the presence of GPI-anchored sequences and signal peptide cleavage sites in *S. cerevisiae* cellular or vesicle proteins. All values used in these analysis were obtained in this study, except those related to GPI-anchored sequences in cellular fractions (ψ) [29].

**Figure 4:**
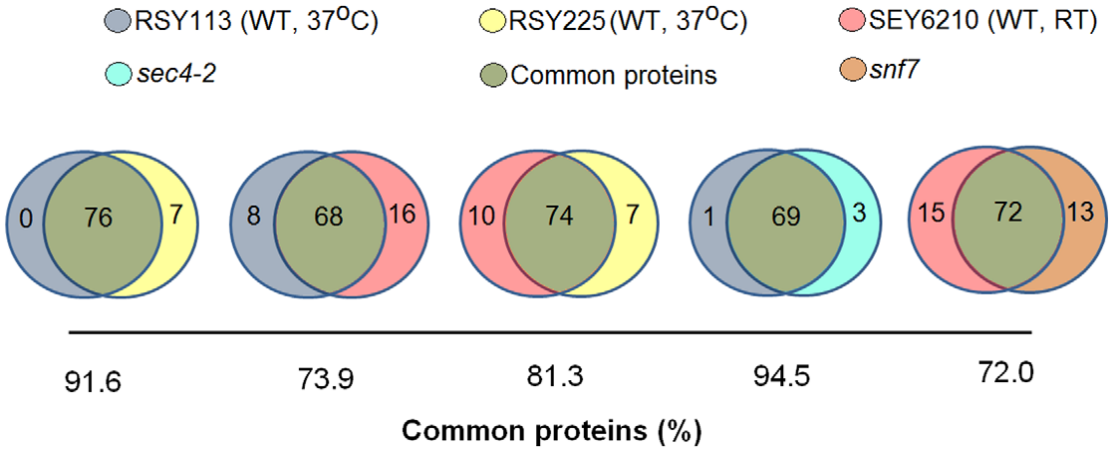
Venn diagrams showing similarities of protein composition in vesicle fractions from WT (RSY113 and SEY6210 strains) and mutant cells (*sec4-2* and *snf7* mutants).

**Figure 5:**
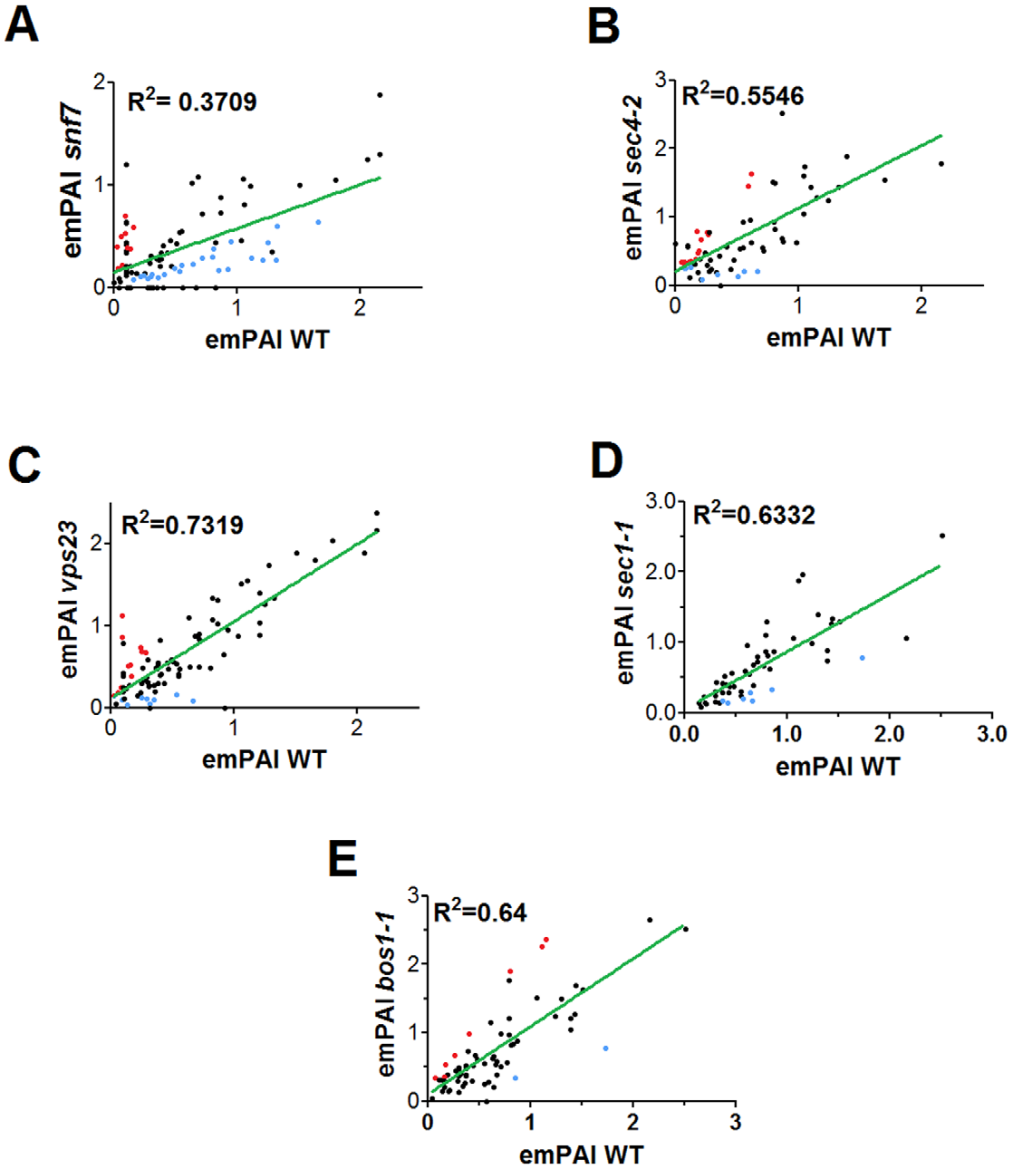
Correlation analysis of the relative variation of protein abundance in vesicle fractions from WT (WT) or mutant *S. cerevisiae* cells. emPAI values for each parental strain were plotted against the values obtained for yeast mutant fractions analyzed by proteomics. *SEC* or MVB-related mutants included *snf7* (A), *sec4-2* (B), *vps23* (C), *sec1-1* (D) and *bos1-1* (E). Lower *R* ^2^ values suggest greater alterations in relative protein distribution in vesicles from the mutants, in comparison to WT cells. Proteins whose abundance was increased in vesicle fractions from mutant cells are represented by the red spots, whereas proteins that were more abundant in WT fractions are highlighted in blue.

Previous studies with *C. neoformans* and *H. capsulatum* revealed that fungal extracellular vesicles carry proteins with highly diverse functions [8], [14]. Many of these proteins were previously reported to also be components of mammalian exosomes [33], [34], [35]. In our model, proteomic analysis was performed with vesicles obtained from three different WT *S. cerevisiae* cells, including strains RSY113, RSY225, and SEY6210. Four hundred proteins were identified in our analysis, with a FDR of ∼1.6% in protein level. Within this group, those with more than 10 spectra after MS/MS analysis of peptides were used for the analyses shown in Figures 2, 3, 4, and 5 and Tables S1 and S2.

A summary of the proteins found in these three strains is summarized in Table S1 and their detailed characterization is shown in Tables S3 and S4.

The *S. cerevisiae* vesicle proteome had many similarities to the protein profiles observed for *C. neoformans* and *H. capsulatum* [8], [14]. Proteins related to diverse metabolic processes consisted of the most abundant functional class in the *S. cerevisiae* vesicles, as previously described for similar models (Figure 2 and Table S1) [8], [14]. Other classes found in the vesicles included cell organization and biogenesis, transporters, carbohydrate metabolism, stress response, protein biosynthesis, protein degradation, protein transport and sporulation. A few molecules with unknown function were also identified (Figure 2A). Of note, twenty four different proteins required for cell wall modeling were identified, including seven different glucanases and three glucanosyl transferases. Eight different heat shock proteins and other stress-related proteins were also found in the vesicles, as well as peptidases, vacuolar and secretory proteins. Although the protein composition of the *S. cerevisiae* extracellular vesicles was multifaceted in many aspects, most of the proteins identified in these preparations (90%) are putatively functionally connected with one or more molecules within the vesicular proteome (Figure 2B). The proportion of proteins showing a high probability of bearing GPI-anchored was approximately 4% of the total vesicular proteome, a value 4-fold higher than those predicted for the cellular *S. cerevisiae* proteome [29]. In addition, prediction of the presence of signal peptide cleavage sites revealed that vesicle fractions also concentrated proteins targeted to the endoplasmic reticulum and the secretory pathway (Figure 3).

Profiles of protein composition of the *S. cerevisiae* vesicles were very similar in different WT cells, as inferred from the high percentage of vesicular proteins shared by distinct strains (Figure 4). The variation range (5–25%) in protein identification observed between different runs of the same sample is within the acceptable range previously reported [36], [37]. Analysis of vesicles obtained from strains RSY113 and RSY225, which were both cultivated at 37°C, revealed that more than 90% of the proteins identified were common to both preparations. These values were also high even when these strains were compared with SEY6210 cells, which were cultivated at room temperature. Proteomic analysis was also performed in vesicle fractions from *S. cerevisiae* secretion mutants (Table S3). Remarkably, most of the proteins found in WT cells were detected in the mutant fractions. The qualitative analysis shown in Figure 4 revealed that most of the proteins found in WT cells were also present in both *sec4-2* and *snf7* mutants, suggesting that the protein compositions were not severely affected by mutations in the secretion genes studied here. Aiming at a more reliable data interpretation, we included in this analysis vesicle proteomics of additional secretion-related systems, which comprised mutations in *SEC1*, *BOS1* (post-Golgi secretion) and *VPS23* (MVB formation) genes. The results obtained were very similar to those detailed in Figure 4 (data not shown).

### Protein abundance in *S. cerevisiae* vesicles is affected by mutations in *SEC* and MVB-related genes

Based on the fact that vesicle protein composition was barely affected by mutations in *SEC4*, *SEC1*, *BOS1*, *VPS23*, and *SNF7* genes, we evaluated whether the relative abundance of vesicle proteins would be modified in cells bearing defects in expression of the related proteins. These analyses were based on semi-quantitative determinations of the abundance of each vesicle protein [26], [27], the same approach used before to describe the proteomic composition of different compartments of the secretory pathway [38]. These methods are based on the fact that the most abundant proteins have a higher coverage by LC-MS/MS. For these analyses we only considered the 127 proteins with at least 10 spectra on one of the samples (Table S5). Within this group, seventy three proteins (57.5%) had changes in their abundance in at least one of the yeast mutants relative to the wild type strain. In the *SEC* mutant group, the abundance of 24 vesicular proteins increased, whereas 11 decreased. Analysis of the *VPS* group of mutants revealed that vesicular protein abundance increased in 14 proteins and decreased in 33. Notably, the collective analysis of graphs correlating emPAI values in vesicle proteins from WT cells with those from mutant strains (Figure 5) demonstrated that the most expressive changes in protein abundance were observed in the *snf7* mutant, followed by *sec4-2*, *sec1-1*, *bos1-1* and *vps23* cells. Individual analysis of protein abundance revealed that the greatest changes (5-fold increase or decrease in comparison to protein abundance in fractions from WT cells) were observed in the *VPS* gene family (Table S5). This analysis revealed an increased abundance of plasma membrane H+-ATPases and mannosyltransferases in the *vps* mutants, with a parallel decrease of glucanases and cyclophilin. These results, which were essentially confirmed by spectral count (data not shown), suggest that vesicle formation is probably affected by multiple elements of the secretory apparatus.

### Mutations in genes involved in either conventional or unconventional secretion affect *S. cerevisiae* extracellular vesicles

**Figure 6:**
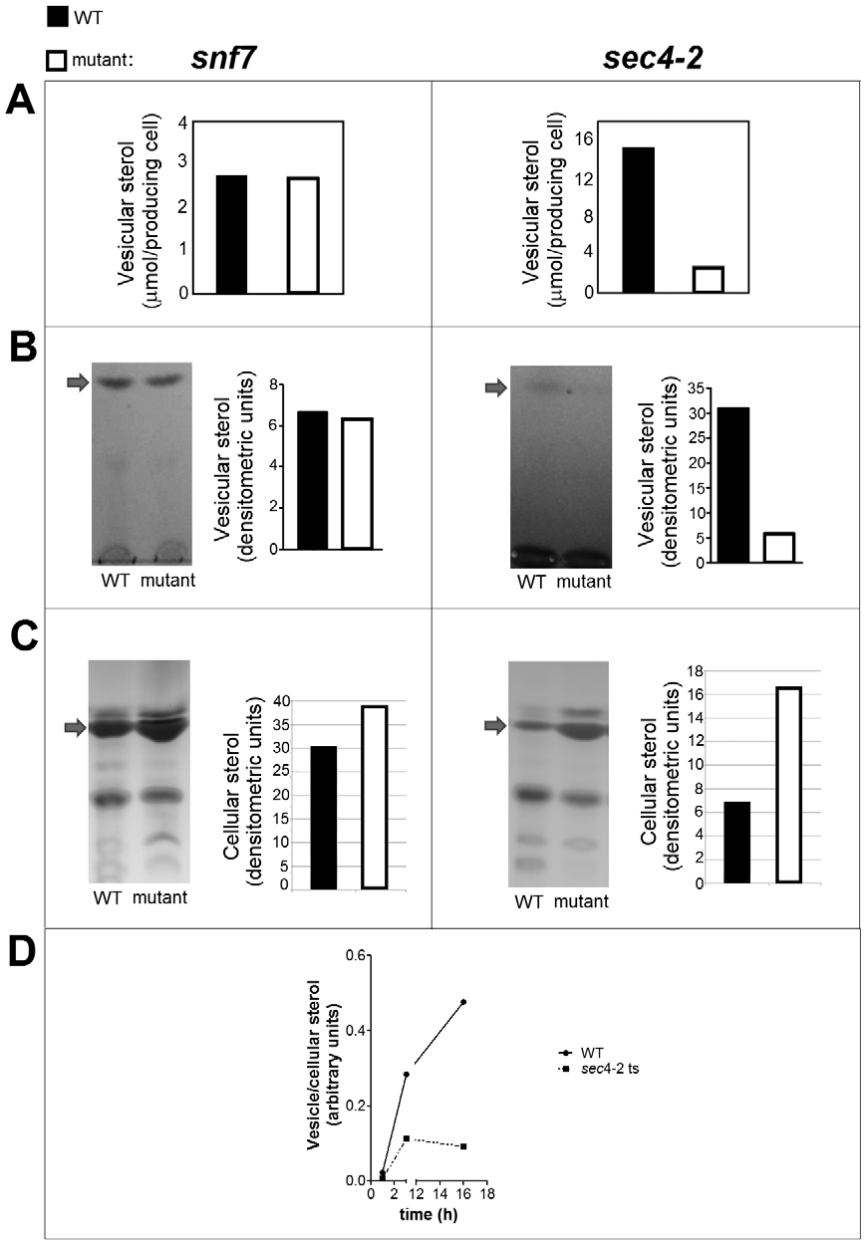
Sterol analysis in vesicle and cellular fractions obtained from yeast cells. A–B. Indirect sterol-based vesicle quantification of fractions obtained from cultures of WT (WT) or mutant cells. The sterol content was determined by fluorimetric methods (A) or by densitometric analysis of bands obtained after HPTLC separation (B). Comparative analysis of the sterol content in vesicle fractions obtained from WT or mutant cells suggested that the *sec4-2* mutant has a defective release of extracellular vesicles. This supposition was supported by chromatographic analysis in association with densitometry of cellular (C) or vesicle (D) fractions obtained from yeast cultures. The *sec4-2* mutant, in contrast to the *snf7* mutant, showed intracellular accumulation of sterols (C) and a lower kinetics of release of sterol-containing vesicles (D). Arrows indicate the migration of an ergosterol standard in TLC plates. Results are representative of three independent analyses showing similar results.

The differences in protein composition led us to consider the possibility that vesicle secretion was somehow altered in the *S. cerevisiae* mutants. Due to the well known characteristic of intracellular vesicle accumulation in the *sec4-2* mutant of *S. cerevisiae* and in the *sav1* mutant of *C. neoformans* [17], [19], [32], we first quantified vesicle release in these cells and in the *snf7* mutant by measuring the content of sterols in cellular and vesicle fractions by different methods. Fluorimetric analysis (Figure 6A) revealed that the sterol content in vesicle fractions from strains SEY6210 and *snf7* were very similar, whereas a large decrease in sterol detection was observed in the mutant *sec4-2*, in comparison to its parental strain (RSY113). These results were essentially confirmed by chromatographic analysis (Figure 6B–C), which showed that, in comparison to WT cells, the *sec4-*2 mutant showed intracellular accumulation of sterols and reduced detection of extracellular vesicular sterols. Similar results were observed for the *sec1.1* mutant (data not shown). The distribution of vesicular and cellular sterols was apparently not significantly affected by mutation in the *SNF7* gene.

The decreased vesicle production in the *sec4-2* mutant led us to analyze the kinetics of vesicle release by these cells, in comparison to the WT strain. While the peak of vesicle secretion in the WT strain occurred after 18 h, detection of sterols in vesicle fractions from the mutant strain stagnated after 3 h (Figure 6D). This result confirmed that release of vesicles to the extracellular space was affected by mutation in the *SEC4* gene.

**Figure 7:**
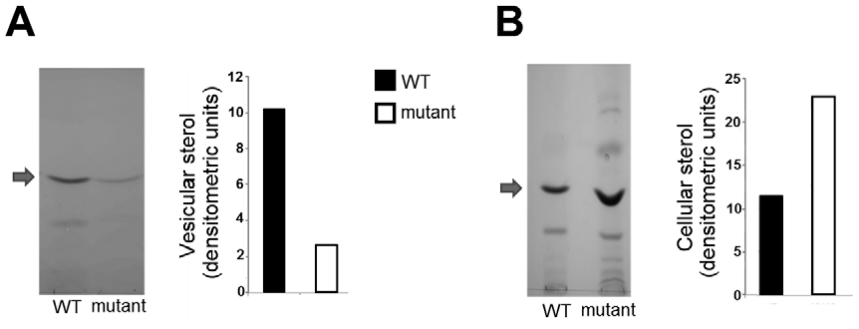
Sterol analysis in vesicle (A) and cellular (B) fractions obtained from WT cells and a mutant strain lacking GRASP expression. Comparative analysis of the sterol content in vesicle fractions obtained from WT or mutant cells suggested that GRASP is involved in the release of extracellular vesicles. Chromatograms and related densitometric analyses are shown. Arrows indicate the chromatographic migration of ergosterol standards. Results are representative of three independent analyses showing similar results.

Due to the clear involvement of elements of the post-Golgi secretory pathway in the kinetics of vesicle release in yeast and the potential involvement of MVB-related pathways with vesicle composition, we asked whether other secretion pathways could be related to vesicular secretion in *S. cerevisiae*. Extracellular vesicle fractions from WT cells and a mutant lacking expression of GRASP, which participates in secretory mechanisms connecting early endosomal compartments and the MVB sorting pathway [39], were analyzed. As observed for the *sec4.2* mutant, yeast cells lacking GRASP expression had significantly reduced sterol content in vesicle fractions and increased intracellular sterol accumulation (Figure 7). This result, which suggests the participation of an unconventional secretion pathway in vesicle production, is in agreement with the fact that approximately 75% of vesicular proteins have no signal peptide sequences for ER translation (Figure 3).

## Discussion

The production of extracellular vesicles by yeast cells has now been reported for at least 6 different fungal species [8], [10], [11], [13], [14], [15], [16]. Fungal extracellular vesicles are believed to function as carriers of distinct molecules to the extracellular space which, in the case of pathogens, includes a wide range of virulence factors, including polysaccharides, pigments, and lipids [8], [10], [11], [13], [14]. As described for mammalian exosomes [40], the vesicular transport in fungi may even include the release of nucleic acids to the extracellular milieu [41]. The secretion of such a complex array of different molecules is expected to impact the interaction of fungal cells with their hosts. In fact, extracellular vesicles isolated from the yeast pathogen *C. neoformans* were recently reported to modulate macrophage functions [42].

Vesicular traffic in eukaryotes is a complex and multifunctional cellular mechanism. Intracellular vesicles are required for the traffic of proteins destined for secretion, in pathways that require their movement from the endoplasmic reticulum to the Golgi complex and then, via the *trans-*Golgi network, to the cell surface [5], [19], [43]. These vesicles are expected to fuse with the plasma membrane releasing their cargo. Vesicular secretion can also involve exosomes, which originate from intracellular MVBs. These organelles derive from endosomes, which form internal vesicles that are released to the extracellular milieu as exosomes upon plasma fusion with the plasma membrane [3]. Yeast extracellular vesicles have been generally termed ‘fungal exosomes’ [9], [14], [16], but different studies suggest that they are linked to elements of the conventional post-Golgi secretory pathway [16], [17], [20]. It remains unknown, nevertheless, whether the formation of MVBs is related to the biogenesis of fungal extracellular vesicles.

*C. neoformans* represents the yeast cell model that extracellular vesicles have been investigated in most detail. This species appears to use *trans-*cell wall vesicular transport to release its major capsular polysaccharide [15], [16], [17], [20], a high molecular weight polysaccharide known as glucuronoxylomannan (GXM). Although it has been suggested that *C. neoformans* produces MVB- and exosome-like structures [14], [44], [45], the vesicular traffic of GXM apparently requires homologues of *SEC4* and *SEC6* genes [16], [17], suggesting that the export of polysaccharide-containing vesicles in these cells requires events of the conventional post-Golgi secretory apparatus. The events required for the biogenesis of extracellular vesicles in these cells are unclear, but the complex and variable morphology of extracellular cryptococcal vesicles analyzed by TEM [14] strongly suggests that the fractions usually analyzed in studies on fungal extracellular vesicles include mixed populations of diverse cellular origin.

Although *C. neoformans* was the species that led to discovery of extracellular vesicles, this fungus may not be the ideal system at this time for genetic dissection of vesicular physiology because the copious extracellular polysaccharide hinders several analytic approaches such as mass spectrometry and genetic tools remain more difficult to use relative to other model fungi. Consequently, we turned our attention to *S. cerevisiae*, where we similarly detected extracellular vesicles in culture supernatants by TEM [8]. Consequently we took advantage of the availability of *S. cerevisiae* secretion mutants and characterized their extracellular vesicles, aiming to identify key elements required for the generation of these compartments in yeast cells. Two major prototypes were used in our study, based on previous literature observations. *Snf7*, a mutant strain with defective MVB formation [31], was selected as a candidate to evaluate whether exosome formation was related to extracellular vesicles in yeast cells. The *sec4-2* mutant was selected as the prototype mutant to evaluate whether events of the post-Golgi conventional secretion were required for the release of fungal extracellular vesicles, given a recent report that the orthologue of *SEC4* in *C. neoformans* is required for the export of polysaccharide-containing vesicles [17]. Using the same rationale, some of the experiments performed in this study included mutant cells with related defects in post-Golgi secretion mechanisms (*sec1-1* and *bos1-1*) and MVB biogenesis (*vps23*) [19], [46], [47].

As determined in this work and in a previous study [11], the diameter of fungal extracellular vesicles ranged from 50 to 500 nm. These dimensions contrast with the fact that extracellular vesicles in other models are in a diameter range lower than 100 nm [3]. Different studies, however, have demonstrated that larger membrane structures (300–500 nm in size) are the vehicles responsible for long distance, ER-to-Golgi and *trans-*Golgi to plasma membrane transport of secretory molecules (reviewed in [48]). Estimation of the diameter of cell wall pores in yeast cells revealed values in the range of 200 to 400 nm [49], which would theoretically permit the release of vesicles of different sizes to the extracellular space. These observations and the high variability in the morphology of fungal extracellular vesicles [14] support the hypothesis that the vesicle populations originate from compartments of distinct biogenesis, which could involve both Golgi- and exosome-derived pathways.

Proteomic analysis of yeast extracellular vesicles revealed a complex composition, as described for mammalian exosomes and other fungal vesicles [14], [33], [34], [35]. Several cytoplasmic proteins with no apparent relation with secretory processes were observed in the vesicle proteome, providing another parallel with mammalian exosomes. Sorting of cytosolic proteins into exosomes is normally explained by a random engulfment of small portions of cytosol during the inward budding process of MVBs [3]. Of note, many cell wall-degrading enzymes were found in the *S. cerevisiae* vesicles, consistent with a prior notion that the passage of vesicles through the cell wall could require hydrolysis of structural components [8], [9], [12]. These enzymes were present in all fractions analyzed in this study. Protein composition was consistently similar in all vesicle fractions analyzed, providing confidence in the validity of the conclusion that vesicle proteins include many different functional classes.

Most of the proteins found in the yeast vesicle proteome were potentially associated other molecules identified in the vesicular protein collection. More precisely, bioinformatics analyses suggested that at least 219 protein-protein interactions were observed within the vesicle proteome. Some of these interactions were in fact expected. For example, YGR032W and YLR342W (glucan synthases) were associated, and they are in the same functional class. Apparently unrelated proteins, however, were also potentially interacting. For instance, YER103W is a heat shock protein that plays a role in protein-membrane targeting and translocation. We found that the molecule interacted with other vesicular heat shock proteins, but also with the metabolic enzyme glyceraldehyde-3-phosphate dehydrogenase [50], [51], [52]. Similarly, YLR249W is a translational elongation factor that interacted with four other elongation factors and two heat shock proteins, but also with pyruvate decarboxylase, phosphoglucose isomerase and alcohol dehydrogenase [53], [54], [55], [56]. These observations illustrate the complexity of the protein composition of fungal extracellular vesicles, as well as the difficulties in unraveling their biosynthetic steps.

Vesicular fractions had greater concentrations of secretory and GPI-anchored proteins in comparison to intact *S. cerevisiae* cells. This observation suggests that the unconventional mechanism of protein secretion by vesicle release includes elements of the conventional secretory pathway. Furthermore, the fact that sequences potentially containing GPI anchors and signals for ER targeting are enriched in vesicle fractions confirms previous observations that fungal extracellular vesicles are actively secreted rather than released by dead cells, since they concentrate secretion-related proteins.

Our semi-quantitative analysis of protein abundance in yeast vesicles strongly suggests that fungal vesicles include compartments related to the MVB-derived pathway of exosome formation. Although vesicle release was similar in WT cells and in the *snf7* mutant, the abundance of 35 proteins was modified in the mutant vesicles, suggesting that defects in MVB formation also affect extracellular vesicles in fungi. In fact, the greatest differences in protein abundance in WT/mutant systems observed in this study involved *vps* mutants and, particularly, *snf7* cells. In vesicle fractions from these mutants, proteins with the greatest levels of increase in relative abundance consisted of two related plasma membrane proton ATPases (YGL008C and YPL036W; Pma1p and Pma2p, respectively) and two Golgi mannosyltransferases (YDR483W and YBR199W). The mannosyltransferases YDR483W and YBR199W are known to interact with other Golgi proteins, which include, respectively, members of the exocyst complex and t-SNAREs required for vesicular transport [57], [58]. Similarly, Pma1p and its isoform Pma2p interact with several elements of Golgi-associated pathways of cellular traffic, including Ric1p, a protein involved in retrograde transport to the cis-Golgi network, and Vps29p, which is essential for endosome-to-Golgi retrograde transport [59], [60]. Therefore, we speculate that mutations in the *VPS* genes could led to the activation of compensatory mechanisms of Golgi-associated traffic, which could explain the increased abundance of Golgi-related proteins in vesicles from *snf7* and *vps23* cells. On the other hand, vesicles from the *snf7* mutants had significantly decreased levels of a protein of unknown function, two glucanases and cyclophilin. The functional implication of each of these individual changes in protein abundance is unknown, but the possibilities are numerous. For instance, cyclophilin is supposed to interact with 34 different proteins in *S. cerevisiae*, including elements of the secretory apparatus [60] and cell wall architecture [61]. These observations illustrate the fact that vesicular proteins with no apparent connections with the secretory process may be directly or indirectly linked to vesicle biogenesis.

Our results show that mutation of the *SEC4* gene is associated with a delay in vesicle release to the extracellular space. This result is supportive and consistent with previous reports that a *sec4-2* mutant of *S. cerevisiae* and a similar mutant in *C. neoformans* accumulate intracellular vesicles [17], [18]. This observation supports the view that the extracellular vesicles observed in fungal cells may not be conventional exosomes, as suggested in independent studies [14], [16], [17]. Nevertheless, it remains unknown how post-Golgi vesicles, which are expected to fuse with the plasma membrane to release their cargo, would leave the cell wall. Different reports, however, suggest that not all secretory vesicles fuse with the plasma membrane (reviewed in [48]), which supports a prior study with a *sec6 C. neoformans* mutant [16] and our current observations with *S. cerevisiae* strains showing that post-Golgi secretion events are required for the release of extracellular vesicles.

Remarkably, vesicle release was not completely abrogated in any of the mutants analyzed in this study. This observation could indicate that multiple cellular pathways are required for formation of fungal extracellular vesicles, including elements of non-conventional secretory mechanisms. In fact, in our study, mutant cells lacking expression of GRASP, which is required for unconventional secretion of an acyl coenzyme A–binding protein in *S. cerevisiae* [39] and *Dictyostelium discoideum* [4], showed a decreased content of extracellular vesicles, in comparison with WT cells. This observation may be related to the fact that acyl coenzyme A–binding protein plays an important role in the cellular distribution of sphingolipids [62], which are important structural components of fungal extracellular vesicles [15]. GRASP is involved in secretory mechanisms that require autophagy genes, early endosomal compartments, and MVBs [39]. The decreased vesicular release by the GRASP mutant reinforces the importance of Golgi components in extracellular vesicle formation and supports the notion that extracellular release of vesicles in fungi is a multifactorial cellular event of high complexity, and possibly involves considerable redundancy. We cannot rule out the possibility, however, that the collection of mutations analyzed in our study are affecting different types of vesicles, since the methods currently used for vesicle purification do not discriminate between vesicles of different origins, resulting in heterogeneous preparations.

Our current results together with previous reports suggest agreement with the fact that endosomes and MVBs can be connected to the *trans-*Golgi secretory pathway [3], which could directly affect the formation of fungal extracellular vesicles. In summary, after analysis of eight different *S. cerevisiae* strains, our results indicate that both MVB- and Golgi-derived cellular pathways affect the formation and release of extracellular vesicles by fungal cells. We believe our observations with *S. cerevisiae* extracellular vesicles will contribute to the understanding of a complex event in the biology of yeast cells. Since yeast extracellular vesicles in pathogens are presumably linked to fungal virulence and the ability of fungal cells to modulate the host immunity, these results could also be of use in the design of pathogenic models aiming at the elucidation of the role of secretion events in fungal virulence.

## Supporting Information

Table S1 Proteomic analysis of S. cerevisiae extracellular vesicles*.
(0.16 MB DOC)

Table S2 Changes in protein abundance in vesicle fractions from secretion mutants of S. cerevisiae, in comparison to fractions from WT cells.
(0.09 MB DOC)

Table S3 All identified proteins.
(0.18 MB XLS)

Table S4 Detailed information of peptide identification.
(0.72 MB XLS)

Table S5 emPAI calculation and spectral count for the 128 proteins used for the semi-quantitative analysis.
(0.08 MB XLS)
