# Supplementary material for: Characterization of Yeast Extracellular Vesicles: Evidence for the Participation of Different Pathways of Cellular Traffic in Vesicle Biogenesis
Source: PLoS One. 2010 Jun 14;5(6):e11113. doi: 10.1371/journal.pone.0011113 (PMC2885426; doi:10.1371/journal.pone.0011113)
Supplement: Table S1 — Proteomic analysis of S. cerevisiae extracellular vesicles*. (0.16 MB DOC) [file pone.0011113.s001.doc]

| **Protein** | **Description** | **Cellular distribution** |
| --- | --- | --- |
| metabolic process | | |
| YLR134W | Pdc5p, minor isoform of pyruvate decarboxylase | Nucleus, cytoplasm |
| YGR087C | Pdc6p, minor isoform of pyruvate decarboxylase | Cytoplasm |
| YKL152C | Gpm1p, tetrameric phosphoglycerate mutase | Cytosol, mitochondria |
| YML028W | Tsa1p, ubiquitous housekeeping thioredoxin peroxidase | Cytoplasm |
| YPR145W | Asn1p, asparagine synthetase | Cytoplasm |
| YLR303W | Met17p, methionine and cysteine synthase | Cytoplasm |
| YJL153C | Ino1p, inositol 1-phosphate synthase | Cytoplasm |
| YDR304C | Cpr5p, peptidyl-prolyl cis-trans isomerase (cyclophilin) of the endoplasmic reticulum | Endoplasmic reticulum, cytoplasm |
| YLR299W | Ecm38p, gamma-glutamyltranspeptidase | Cytoplasm, plasma membrane |
| YDR483W | Kre2p, alpha1,2-mannosyltransferase of the Golgi involved in protein mannosylation | Golgi apparatus, plasma membrane, Golgi stack |
| YGR192C | Tdh3p, glyceraldehyde-3-phosphate dehydrogenase | Cytoplasm, mitochondria, cell wall |
| YLR109W | Ahp1p, thiol-specific peroxiredoxin | Peroxisome, plasma membrane, cytoplasm |
| YGR180C | Rnr4p, ribonucleotide-diphosphate reductase (RNR), small subunit | Ribonucleoside-diphosphate reductase, nucleus, cytoplasm |
| YHR183W | Gnd1p, 6-phosphogluconate dehydrogenase | Mitochondria, cytoplasm |
| YHR179W | Oye2p, Widely conserved NADPH oxidoreductase containing flavin mononucleotide (FMN), may be involved in sterol metabolism | Nucleus, mitochondria, cytoplasm |
| YGL234W | Ade5,7p, Bifunctional enzyme of the 'de novo' purine nucleotide biosynthetic pathway | Cytoplasm |
| YOL011W | Plb3p, Phospholipase B (lysophospholipase); hydrolyzes phosphatidylinositol and phosphatidylserine | Extracellular region, plasma membrane |
| YLR245C | Cdd1p, Cytidine deaminase; catalyzes the modification of cytidine to uridine | Nucleus, cytoplasm |
| YDL124W | Ydl124wp, NADPH-dependent alpha-keto amide reductase | Nucleus, cytoplasm |
| YML126C | Erg13p, 3-hydroxy-3-methylglutaryl-CoA (HMG-CoA) synthase | Endoplasmic reticulum, mitochondria |
| YDR050C | Tpi1p, Triose phosphate isomerase, abundant glycolytic enzyme. | Cytosol, mitochondria,  Cytoplasm |
| YLR044C | Pdc1p, Major of three pyruvate decarboxylase isozymes, key enzyme in alcoholic fermentation. | Nucleus, cytoplasm |
| YGL202W | Aro8p, Aromatic aminotransferase I | Cytoplasm |
| YDR044W | Hem13p, Coproporphyrinogen III oxidase, catalyzes the sixth step in the heme biosynthetic pathway | Mitochondrial inner membrane |
| YEL047C | Yel047cp, Soluble fumarate reductase | Cytosol, ribosome, mitochondria |
| YKL060C | Fba1p, Fructose 1,6-bisphosphate aldolase | Cytosol, mitochondria, cytoplasm |
| YBR199W | Ktr4p, Putative mannosyltransferase involved in protein glycosylation; member of the KRE2/MNT1 mannosyltransferase family | Golgi apparatus, integral to membrane, membrane |
| YBR196C | Pgi1p, Glycolytic enzyme phosphoglucose isomerase | Cytosol, mitochondria |
| YCR012W | Pgk1p, 3-phosphoglycerate kinase; key enzyme in glycolysis and gluconeogenesis | Cytosol, mitochondria, cytoplasm |
| YJL052W | Tdh1p, Glyceraldehyde-3-phosphate dehydrogenase, isozyme 1, involved in glycolysis and gluconeogenesis | Lipid particle, Cell wall, cytosol, mitochondria, cytoplasm |
| YJR009C | Tdh2p, Glyceraldehyde-3-phosphate dehydrogenase, isozyme 2, involved in glycolysis and gluconeogenesis | Lipid particle, Cell wall, cytosol, mitochondria, cytoplasm |
| YAL038W | Cdc19p, Pyruvate kinase | Cytosol |
| YMR303C | Adh2p, Glucose-repressible alcohol dehydrogenase II | Cytoplasm |
| YGR254W | Eno1p, Enolase I, a phosphopyruvate hydratase | Mitochondria, vacuole, cytoplasm |
| YOR133W | Eft1p, Elongation factor 2 (EF-2), catalyzes ribosomal translocation during protein synthesis | Ribosome |
| YER091C | Met6p, Cobalamin-independent methionine synthase, involved in amino acid biosynthesis | Cytoplasm |
| YCL043C | Pdi1p, Protein disulfide isomerase, multifunctional protein resident in the endoplasmic reticulum lumen, essential for the formation of disulfide bonds in secretory and cell-surface proteins | Endoplasmic reticulum, endoplasmic reticulum lumen |
| YAL012W | Cys3p, Cystathionine gamma-lyase, catalyzes one of the two reactions involved in the transsulfuration pathway that yields cysteine from homocysteine | Cytoplasm |
| YDR533C | Hsp31p, Possible chaperone and cysteine protease | Soluble fraction |
| YDL045C | Fad1p, Flavin adenine dinucleotide (FAD) synthetase | Cytoplasm |
| YJR148W | Bat2p, Cytosolic branched-chain amino acid aminotransferase | Nucleus, cytoplasm |
| YHR057C | Cpr2p, Peptidyl-prolyl cis-trans isomerase (cyclophilin), catalyzes the cis-trans isomerization of peptide bonds N-terminal to proline residues; has a potential role in the secretory pathway | Cellular component unknown |
| YOR099W | Ktr1p, Alpha-1,2-mannosyltransferase involved in O- and N-linked protein glycosylation | Golgi apparatus,  integral to membrane, membrane, Golgi stack |
| YJR139C | Hom6p, Homoserine dehydrogenase (L-homoserine:NADP oxidoreductase), dimeric enzyme that catalyzes the third step in the common pathway for methionine and threonine biosynthesis; | Nucleus, cytoplasm |
| YHR008C | Sod2p, Mitochondrial superoxide dismutase, protects cells against oxygen toxicity | Mitochondrial matrix, mitochondria |
| YER043C | Sah1p, S-adenosyl-L-homocysteine hydrolase | Cytoplasm |
| YNL117W | Mls1p, Malate synthase, enzyme of the glyoxylate cycle, involved in utilization of non-fermentable carbon sources | Peroxisomal matrix, glyoxysome, cytoplasm |
| YKL216W | Ura1p, Dihydroorotate dehydrogenase, catalyzes the fourth enzymatic step in the de novo biosynthesis of pyrimidines | Extrinsic to membrane, cytoplasm |
| YOR375C | Gdh1p, NADP(+)-dependent glutamate dehydrogenase | Nucleus, cytoplasm |
| YHR174W | Eno2p, Enolase II, a phosphopyruvate hydratase activity | Soluble fraction, vacuole, mitochondria |
| YHR208W | Bat1p, Mitochondrial branched-chain amino acid aminotransferase | Mitochondrial matrix, mitochondria |
| YJL130C | Ura2p, Bifunctional carbamoylphosphate synthetase (CPSase)-aspartate transcarbamylase (ATCase), catalyzes the first two enzymatic steps in the de novo biosynthesis of pyrimidines | Mitochondria, cytoplasm,  integral to membrane |
| YAR071W | Pho11p, One of three repressible acid phosphatases, a glycoprotein that is transported to the cell surface by the secretory pathway | Extracellular region |
| YBR011C | Ipp1p, Cytoplasmic inorganic pyrophosphatase (PPase) | Cytosol |
| YJL026W | Rnr2p, Ribonucleotide-diphosphate reductase (RNR), small subunit | Nucleus, cytoplasm |
| YGR256W | Gnd2p, 6-phosphogluconate dehydrogenase (decarboxylating), catalyzes an NADPH regenerating reaction in the pentose phosphate pathway | Cytosol |
| YOL086C | Adh1p, Alcohol dehydrogenase, fermentative isozyme | Cytosol |
| YKL157W | Ape2p, Zinc-dependent metallopeptidase yscII | Mitochondria, cell wall-bounded periplasmic space, cytoplasm |
| cell organization and biogenesis | | |
| YPL053C | Ktr6p, Probable mannosylphosphate transferase involved in the synthesis of core oligosaccharides in protein glycosylation pathway | Membrane fraction, integral to membrane |
| YGR032W | Gsc2p, Catalytic subunit of 1,3-beta-glucan synthase, involved in formation of the inner layer of the spore wall | Prospore membrane, 1,3-beta-glucan synthase complex, membrane fraction, integral to membrane, actin cap |
| YLR342W | Fks1p, Catalytic subunit of 1,3-beta-D-glucan synthase, involved in cell wall synthesis and maintenance; localizes to sites of cell wall remodeling | 1,3-beta-glucan synthase complex, integral to membrane, actin cortical patch, actin cap, mitochondria |
| YLR355C | Ilv5p, Acetohydroxyacid reductoisomerase, mitochondrial protein involved in amino acid biosynthesis, also required for maintenance of mitochondrial DNA | Mitochondria, mitochondrial nucleoid |
| YMR307W | Gas1p, Beta-1,3-glucanosyltransferase, required for cell wall assembly; localizes to the cell surface via a glycosylphosphatidylinositol (GPI) anchor | Cell wall, plasma membrane, mitochondria |
| YGR282C | Bgl2p, Endo-beta-1,3-glucanase, major protein involved in cell wall maintenance | Cell wall |
| YDR055W | Pst1p, Cell wall protein that contains a putative GPI-attachment site; secreted by regenerating protoplasts; up-regulated by cell wall damage | Cell wall, Plasma membrane |
| YLR249W | Yef3p, Translational elongation factor 3, stimulates the binding of aminoacyl-tRNA (AA-tRNA) to ribosomes | Ribosome |
|
| YDR261C | Exg2p, Exo-1,3-beta-glucanase, involved in cell wall beta-glucan assembly; may be anchored to the plasma membrane via a glycosylphosphatidylinositol (GPI) anchor | Cell wall, membrane |
| YBR078W | Ecm33p, GPI-anchored protein of unknown function, has a possible role in apical bud growth | Membrane fraction, Cell wall, mitochondria |
| YJR105W | Ado1p, Adenosine kinase, may be involved in recycling of adenosine | Nucleus, cytoplasm |
| YLR300W | Exg1p, Major exo-1,3-beta-glucanase involved in cell wall beta-glucan assembly | Cell wall |
| YLR121C | Yps3p, Aspartic protease, attached to the plasma membrane via a glycosylphosphatidylinositol (GPI) anchor | Anchored to plasma membrane |
| YMR186W | Hsc82p, Cytoplasmic chaperone of the Hsp90 family, redundant in function and nearly identical with Hsp82p | Mitochondria, cytoplasm |
| YFL039C | Act1p, Actin, structural protein involved in cell polarization, endocytosis, and other cytoskeletal functions | Cytoplasm |
| YLL026W | Hsp104p, Heat shock protein that cooperates to refold and reactivate previously denatured, aggregated proteins; responsive to stresses including: heat, ethanol, and sodium arsenite | Nucleus, cytoplasm |
| YPL106C | Sse1p, ATPase that is a component of the heat shock protein Hsp90 chaperone complex | Cytoplasm |
| Transporters | | |
| YPL036W | Pma2p, Plasma membrane H+-ATPase, isoform of Pma1p, regulator of cytoplasmic pH and plasma membrane potential | Mitochondria, plasma membrane, Integral to membrane |
| YGL253W | Hxk2p, Hexokinase isoenzyme 2 that catalyzes phosphorylation of glucose in the cytosol | Cytosol, mitochondria, nucleus |
| YCR053W | Thr4p, Threonine synthase, conserved protein that catalyzes formation of threonine | Nucleus, cytoplasm |
| YPR080W | Tef1p,Translational elongation factor EF-1 alpha; functions in the binding reaction of aminoacyl-tRNA (AA-tRNA) to ribosomes | Eukaryotic translation elongation factor 1 complex, ribosome, mitochondria |
| YJR048W | Cyc1p, Cytochrome c, isoform 1; electron carrier of the mitochondrial intermembrane space | Mitochondrial intermembrane space  Mitochondrial respiratory chain |
| YGL008C | Pma1p, Plasma membrane H+-ATPase, major regulator of cytoplasmic pH and plasma membrane potential | Lipid raft, mitochondria, plasma membrane |
| YFR053C | Hxk1p, Hexokinase isoenzyme 1 | Cytosol |
| YDL046W | Npc2p , Functional homolog of human NPC2/He1 | Vacuole, cell cycle-correlated morphology |
| YEL039C | Cyc7p, Cytochrome c isoform 2; electron carrier of the mitochondrial intermembrane space | Mitochondrial respiratory chain |
| carbohydrate metabolism | | |
| YGL028C | Scw11p , Cell wall protein with similarity to glucanases; may play a role in conjugation during mating | Cell wall |
| YGR279C | Scw4p, Cell wall protein with similarity to glucanases | Cell wall |
| YNR067C | Dse4p, Daughter cell-specific secreted protein with similarity to glucanases, degrades cell wall from the daughter side causing daughter to separate from mother | Extracellular region, Cell wall |
| YMR105C | Pgm2p, Phosphoglucomutase, catalyzes a key step in hexose metabolism | Cytosol, cytoplasm |
| YLR354C | Tal1p, Transaldolase, enzyme in the non-oxidative pentose phosphate pathway | Cytoplasm |
| YMR305C | Scw10p, Cell wall protein with similarity to glucanases; may play a role in conjugation during mating | Endoplasmic reticulum, Cell wall, cytoplasm |
| response to stress | | |
| YMR169C | Ald3p, Cytoplasmic aldehyde dehydrogenase, involved in beta-alanine synthesis | Cytoplasm |
| YGR234W | Yhb1p, Nitric oxide oxidoreductase, flavohemoglobin involved in nitric oxide detoxification; plays a role in the oxidative and nitrosative stress responses | Mitochondrial matrix, cytoplasm, cytosol |
| YDL229W | Ssb1p, Cytoplasmic ATPase that is a ribosome-associated molecular chaperone | Soluble fraction, polysome |
| YNL160W | Ygp1p , Cell wall-related secretory glycoprotein; induced by nutrient deprivation-associated growth arrest and upon entry into stationary phase | Cell wall |
| YNL209W | Ssb2p, Cytoplasmic ATPase that is a ribosome-associated molecular chaperone | Polysome, cytoplasm |
| YPL240C | Hsp82p, Hsp90 chaperone required for pheromone signaling | Cytoplasm |
| Protein biosynthesis | | |
| YLR075W | Rpl10p , Protein component of the large (60S) ribosomal subunit | Cytosolic large ribosomal subunit |
| YBR118W | Tef2p,Translational elongation factor EF-1 alpha; functions in the binding reaction of aminoacyl-tRNA (AA-tRNA) to ribosomes | Eukaryotic translation elongation factor 1 complex, ribosome, cytoplasm |
| YDR012W | Rpl4bp, Protein component of the large (60S) ribosomal subunit | Cytosolic large ribosomal subunit, cytoplasm |
| YDR385W | Eft2p, Elongation factor 2 (EF-2); catalyzes ribosomal translocation during protein synthesis | Ribosome |
| YBR031W | Rpl4ap, N-terminally acetylated protein component of the large (60S) ribosomal subunit | Cytosolic large ribosomal subunit |
| YJR123W | Rps5p, Protein component of the small (40S) ribosomal subunit | Cytosolic small ribosomal subunit |
| protein degradation | | |
| YCL057W | Prd1, Zinc metalloendopeptidase, involved in degradation of mitochondrial proteins and of presequence peptides cleaved from imported proteins | Mitochondrial intermembrane space,  Cytoplasm |
| YMR297W | Prc1p, Vacuolar carboxypeptidase Y (proteinase C), broad-specificity C-terminal exopeptidase involved in non-specific protein degradation in the vacuole; member of the serine carboxypeptidase family; protein coding | Lumen of vacuole with cell cycle-correlated morphology, endoplasmic reticulum, cytoplasm |
| YPL235W | Rvb2p, Essential protein involved in transcription regulation; component of chromatin remodeling complexes | Nucleus, chromatin remodeling complex, SWR1 complex, INO80 complex |
| YFR044C | Dug1p, Probable di- and tri-peptidase; forms a complex to degrade glutathione (GSH) | Ribosome, mitochondria, cytoplasm |
| YGL203C | Kex1p, Protease involved in the processing of killer toxin and alpha factor precursor; cleaves Lys and Arg residues from the C-terminus of peptides and proteins; protein coding | Trans-Golgi network, integral to membrane |
| protein transport | | |
| YJL034W | Kar2p , ATPase involved in protein import into the ER, also acts as a chaperone to mediate protein folding in the ER and may play a role in ER export of soluble proteins | Endoplasmic reticulum lumen, endoplasmic reticulum |
| YLL024C | Ssa2p , ATP binding protein involved in protein folding and vacuolar import of proteins | Membrane of vacuole with cell cycle-correlated morphology, chaperonin-containing T-complex, Cell wall, mitochondria, cytoplasm |
| YBL075C | Ssa3p, ATPase involved in protein folding and the response to stress; plays a role in SRP-dependent cotranslational protein-membrane targeting and translocation; member of the heat shock protein 70 (HSP70) family | Cytosol |
| YAL005C | Ssa1p, ATPase involved in protein folding and nuclear localization signal (NLS)-directed nuclear transport; member of heat shock protein 70 (HSP70) family | Membrane of vacuole with cell cycle-correlated morphology, chaperonin-containing T-complex, Cell wall, nucleus, cytoplasm |
| YER103W | Ssa4p, Heat shock protein that is highly induced upon stress; plays a role in SRP-dependent cotranslational protein-membrane targeting and translocation | Nucleus, cytoplasm |
| Sporulation | | |
| YPL154C | Pep4p, Vacuolar aspartyl protease (proteinase A), required for the posttranslational precursor maturation of vacuolar proteinases; important for protein turnover after oxidative damage | Lumen of vacuole with cell cycle-correlated morphology, mitochondria |
| YDR155C | Cpr1p, Cytoplasmic peptidyl-prolyl cis-trans isomerase (cyclophilin), catalyzes the cis-trans isomerization of peptide bonds N-terminal to proline residues | Histone deacetylase complex, mitochondria, nucleus |
| YER177W | Bmh1p, 14-3-3 protein, major isoform; controls proteome at post-transcriptional level, binds proteins and DNA, involved in regulation of many processes including exocytosis, vesicle transport, Ras/MAPK signaling, and rapamycin-sensitive signaling | Nucleus |
|
| YDR099W | Bmh2p, 14-3-3 protein, minor isoform; controls proteome at post-transcriptional level, binds proteins and DNA, involved in regulation of many processes including exocytosis, vesicle transport, Ras/MAPK signaling, and rapamycin-sensitive signaling | Nucleus |
|
|
| Unknown | | |
| YJL171C | Yjl171cp, GPI-anchored cell wall protein of unknown function; induced in response to cell wall damaging agents and by mutations in genes involved in cell wall biogenesis | Cell wall, mitochondria |
| YNL134C | Ynl134cp, Putative protein of unknown function with similarity to dehydrogenases from other model organisms | Nucleus, cytoplasm |
| YLR178C | Tfs1p, Carboxypeptidase Y inhibitor, phosphatidylethanolamine-binding protein involved in protein kinase A signaling pathway | Membrane of vacuole with cell cycle-correlated morphology, soluble fraction, lumen of vacuole with cell cycle-correlated morphology, cytoplasm |
| YLR179C | Ylr179cp, Protein of unknown function, transcription is activated by paralogous proteins Yrm1p and Yrr1p along with proteins involved in multidrug resistance | Nucleus, cytoplasm |
| YHR138C | Yhr138cp, Putative protein of unknown function; has similarity to Pbi2p; double null mutant lacking Pbi2p and Yhr138p exhibits highly fragmented vacuoles | Cellular component unknown |
| YNL208W | Ynl208wp, Protein of unknown function; may interact with ribosomes, based on co-purification experiments; authentic, non-tagged protein is detected in purified mitochondria | Ribosome, mitochondria |
| YLR414C | Ylr414cp, Putative protein of unknown function; transcriptional induced in response to cell wall damage; YLR414C is not an essential gene | Cellular bud, cytoplasm |
| YDR262W | Ydr262wp, Putative protein of unknown function; induced in response to the DNA-damaging agent MMS; gene expression increases in response to Zymoliase treatment | Vacuole, cell cycle-correlated morphology |
| YOL030W | Gas5p, 1,3-beta-glucanosyltransferase, has similarity to Gas1p; localizes to the cell wall | Membrane fraction, membrane, Cell wall |
| YMR215W | Gas3p, Putative 1,3-beta-glucanosyltransferase, has similarity to Gas1p; localizes to the cell wall | Membrane fraction, membrane, Cell wall |
| YPL225W | Ypl225wp, Protein of unknown function that may interact with ribosomes | Ribosome, cytoplasm |
| YHR215W | Pho12p , One of three repressible acid phosphatases, a glycoprotein that is transported to the cell surface by the secretory pathway; nearly identical to Pho11p | Vacuole, cell cycle-correlated morphology |
|

* Proteins with less than 10 spectra after MS/MS analysis were not listed in this table.
