# Supplementary material for: Characterization of Yeast Extracellular Vesicles: Evidence for the Participation of Different Pathways of Cellular Traffic in Vesicle Biogenesis
Source: PLoS One. 2010 Jun 14;5(6):e11113. doi: 10.1371/journal.pone.0011113 (PMC2885426; doi:10.1371/journal.pone.0011113)
Supplement: Table S2 — Changes in protein abundance in vesicle fractions from secretion mutants of S. cerevisiae, in comparison to fractions from WT cells. (0.09 MB DOC) [file pone.0011113.s002.doc]

| Protein | Mutant | Protein abundance incremental change (%)* |
| --- | --- | --- |
| YPL036W, plasma membrane H+-ATPase, isoform of Pma1p | *snf7 vps23* | 1404.2  528.9 |
| YBR199W, putative mannosyltransferase involved in protein glycosylation | *vps23*  *snf7* | 1135.5  534.2 |
| YDR483W, alpha1,2-mannosyltransferase of the Golgi involved in protein mannosylation | *vps23*  *snf7* | 927.4  757.3 |
| YGL008C, plasma membrane H+-ATPase | *snf7 vps23* | 837.5  318.1 |
| YGR234W, nitric oxide oxidoreductase | *sec4-2* | 680.6 |
| YER091C, cobalamin-independent methionine synthase | *bos1-1*  *sec4-2*  *vps23* | 448.7  448.7  242.7 |
| YBR196C, phosphoglucose isomerase | *sec4-2*  *snf7* | 437.2  23.5 |
| YHR183W, 6-phosphogluconate dehydrogenase | *sec4-2*  *bos1-1*  *snf7* | 387.7  251.1  45.9 |
| YPR145W, asparagine synthetase | *sec4-2* | 385.0 |
| YNL209W, cytoplasmic ATPase; member of the HSP70 family | *sec4-2* | 384.4 |
| YER043C, S-adenosyl-L-homocysteine hydrolase | *snf7*  *vps23* | 352.5  304.9 |
| YLR075W, protein component of the large (60S) ribosomal subunit | *snf7* | 336.1 |
| YCR053W, threonine synthase | *sec4-2*  *snf7* | 325.0  39.5 |
| YDL229W, cytoplasmic ATPase; member of the HSP70 family | *sec4-2* | 323.3 |
| YLR249W, translational elongation factor 3 | *snf7* | 320.9 |
| YBR011C, cytoplasmic inorganic pyrophosphatase | *sec4-2*  *snf7* | 315.8  45.2 |
| YGR087C, minor isoform of pyruvate decarboxylase | *vps23*  *snf7* | 315.8  48.1 |
| YGR180C, ribonucleotide-diphosphate reductase, small subunit | *sec4-2* | 315.8 |
| YHR179W, NADPH oxidoreductase | *bos1-1*  *sec4-2* | 306.9  217.4 |
| YLR354C, transaldolase | *vps23* | 297.8 |
| YJL130C, bifunctional carbamoylphosphate synthetase (CPSase)-aspartate transcarbamylase (ATCase) | *snf7*  *vps23* | 284.4  257.3 |
| YJL153C, inositol 1-phosphate synthase | *sec4-2* | 276.4 |
| YBR078W, GPI-anchored protein of unknown function | *snf7* | 276.2 |
| YDL045C, flavin adenine dinucleotide (FAD) synthetase | *sec4-2* | 274.7 |
| YML126C, 3-hydroxy-3-methylglutaryl-CoA (HMG-CoA) synthase | *vps23* | 267.3 |
| YEL047C, soluble fumarate reductase | *sec4-2* | 267.3 |
| YLR109W, thiol-specific peroxiredoxin | *sec4-2* | 262.4 |
| YGL234W, bifunctional enzyme of the 'de novo' purine nucleotide biosynthetic pathway | *sec4-2* | 262.4 |
| YFR053C, hexokinase isoenzyme 1 | *sec4-2* | 243.4 |
| YKL152C, tetrameric phosphoglycerate mutase | *bos1-1* | 240.7 |
| YJL052W, glyceraldehyde-3-phosphate dehydrogenase | *bos1-1*  *snf7* | 235.4  26.7 |
| YDR533C, possible chaperone and cysteine protease | *vps23* | 229.2 |
| YMR297W, vacuolar carboxypeptidase Y | *vps23*  *sec1-1* | 217.4  46.0 |
| YJR139C, homoserine dehydrogenase | *sec4-2* | 217.4 |
| YLR355C, acetohydroxyacid reductoisomerase | *bos1-1*  *snf7* | 216.2  17.7 |
| YCL043C, protein disulfide isomerase | *sec4-2*  *vps23* | 212.6  12.6 |
| YGR192C, glyceraldehyde-3-phosphate dehydrogenase, isozyme 3 | *bos1-1* | 205.4 |
| YJR009C, glyceraldehyde-3-phosphate dehydrogenase, isozyme 2 | *bos1-1* | 203.8 |
| YOL011W, phospholipase B | *snf7* | 47.5 |
| YGR279C, cell wall protein with similarity to glucanases | *snf7* | 47.3 |
| YJR148W, cytosolic branched-chain amino acid aminotransferase | *snf7* | 47.3 |
| YAL012W, cystathionine gamma-lyase | *vps23* | 47.2 |
| YPL240C, Hsp90 chaperone | *sec4-2* | 46.3 |
| YCR053W, threonine synthase | *sec1-1* | 46.1 |
| YGR254W, enolase I | *snf7* | 45.1 |
| YLR245C, cytidine deaminase | *sec1-1*  *bos1-1* | 44.8  44.8 |
| YGL253W, hexokinase isoenzyme 2 | *snf7* | 44.1 |
| YJR105W, adenosine kinase | *sec1-1* | 43.8 |
| YAL005C, ATPase involved in protein folding and nuclear localization signal, member of HSP70 family | *snf7* | 40.4 |
| YLL024C, ATP binding protein involved in protein folding and vacuolar import of proteins; member of HSP70 family | *snf7* | 40.4 |
| YNR067C, daughter cell-specific secreted protein with similarity to glucanases | *sec4-2* | 40.4 |
| YLR121C, GPI-anchored aspartic protease | *snf7* | 39.1 |
| YPL053C, probable mannosylphosphate transferase involved in the synthesis of core oligosaccharides | *sec1-1*  *bos1-1*  *sec4-2* | 39.1  39.1  25.8 |
| YLR300W, major exo-1,3-beta-glucanase of the cell wall | *snf7* | 38.0 |
| YNL134C, putative protein of unknown function with similarity to dehydrogenases from other model organisms | *snf7* | 38.0 |
| YER177W, 14-3-3 protein, major isoform | *sec4-2* | 37.5 |
| YHR208W, mitochondrial branched-chain amino acid aminotransferase | *vps23* | 36.9 |
| YCR012W, 3-phosphoglycerate kinase | *snf7* | 34.7 |
| YOR375C, NADP(+)-dependent glutamate dehydrogenase | *sec1-1* | 34.7 |
| YBL075C, ATPase involved in protein folding and the response to stress; member of HSP70 family | *snf7* | 34.5 |
| YML028W, ubiquitous housekeeping thioredoxin peroxidase | *snf7* | 34.1 |
| YJL026W, ribonucleotide-diphosphate reductase (RNR), small subunit | *sec4-2* | 31.7 |
| YGR256W, 6-phosphogluconate dehydrogenase | *snf7* | 30.6 |
| YLR134W, minor isoform of pyruvate decarboxylase | *snf7* | 29.6 |
| YKL216W, dihydroorotate dehydrogenase | *snf7*  *vps23* | 28.7  28.7 |
| YAL038W, pyruvate kinase | *sec1-1* | 25.6 |
| YFR044C, probable di- and tri-peptidase | *snf7* | 25.5 |
| YNL117W, malate synthase | *vps23* | 25.5 |
| YJL034W, ATPase involved in protein import into the ER | *vps23* | 23.8 |
| YMR305C, cell wall protein with similarity to glucanases | *snf7* | 22.2 |
| YGR282C, endo-beta-1,3-glucanase | *snf7* | 22.2 |
| YHR138C, protein of unknown function | *snf7* | 19.2 |
| YDR304C, peptidyl-prolyl cis-trans isomerase (cyclophilin) of the endoplasmic reticulum | *vps23* | 13.3 |

(*) Values represent the incremental changes of the abundance of each protein from mutant cells, in comparison with emPAI values obtained for vesicles from WT cells
